# Application of Dithiocarbamates as Potential New Antitrypanosomatids-Drugs: Approach Chemistry, Functional and Biological

**DOI:** 10.3390/molecules24152806

**Published:** 2019-08-01

**Authors:** Johny Wysllas de Freitas Oliveira, Hugo Alexandre Oliveira Rocha, Wendy Marina Toscano Queiroz de Medeiros, Marcelo Sousa Silva

**Affiliations:** 1 Laboratório de Imunoparasitologia, Departamento de Análises Clínicas e Toxicológicas, Centro de Ciências da Saúde, Universidade Federal do Rio Grande do Norte, Natal 59012-570, Brazil; 2 Programa de Pós-graduação em Bioquímica, Centro de Biociências, Universidade Federal do Rio Grande do Norte, Natal 59012-570, Brazil; 3 Programa de Pós-graduação em Ciências Farmacêuticas, Centro de Ciências da Saúde, Universidade Federal do Rio Grande do Norte, Natal 59012-570, Brazil; 4 Global Health and Tropical Medicine, Instituto de Higiene e Medicina Tropical, Universidade Nova de Lisboa, 1800-166 Lisbon, Portugal

**Keywords:** dithiocarbamates, DETC, antiparasitic activity, *Trypanosomatids*, Chagas disease, leishmaniasis, African trypanosomiasis

## Abstract

Dithiocarbamates represent a class of compounds that were evaluated in different biomedical applications because of their chemical versatility. For this reason, several pharmacological activities have already been attributed to these compounds, such as antiparasitic, antiviral, antifungal activities, among others. Therefore, compounds that are based on dithiocarbamates have been evaluated in different in vivo and in vitro models as potential new antimicrobials. Thus, the purpose of this review is to present the possibilities of using dithiocarbamate compounds as potential new antitrypanosomatids-drugs, which could be used for the pharmacological control of Chagas disease, leishmaniasis, and African trypanosomiasis.

## 1. Introduction

The year in which the dithiocarbamic acids were discovered is unknown, but the first report of its use was in the year 1850 by Debus [1]. Dithiocarbamic acids are a class of chemical compounds that are used in a variety of pharmacological applications, such as antiparasitic, antifungal, antiviral, and antitumor activities. Structurally, dithiocarbamates (R2CNS-2) consist of a broad class of 1,1-dithioligand monoacids, in which they can be easily synthesized from a wide variety of compounds [2,3,4,5,6,7].

**Figure 1:**
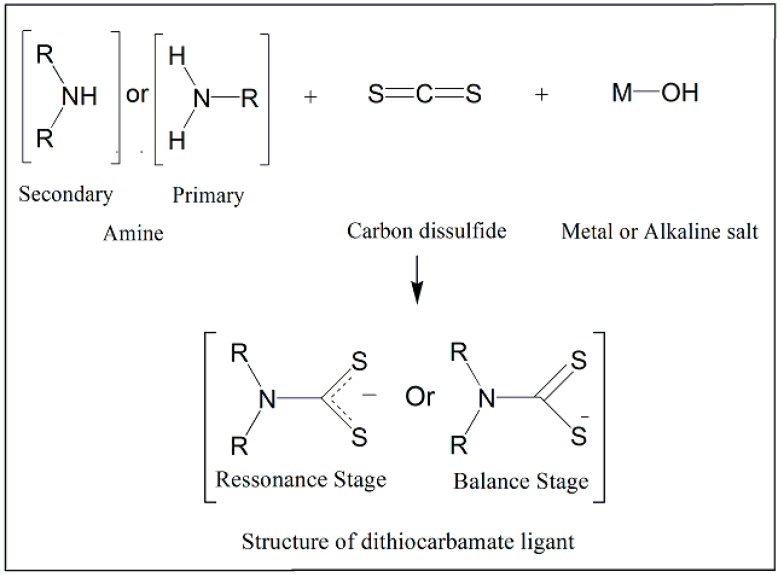
Schematic representation of the chemical synthesis of dithiocarbamates-based compounds.

The dithiocarbamate synthesis has a large variety, depending on the final product. The traditional method is based on the reaction of a primary or secondary amine with carbon disulfide in the presence of metal or alkaline salt, due to low stability of these acids (Figure 1). In this process, the type of amine interferes with the substituents formation differing in mono or diacyl dithiocarbamic, and the type of structure can vary between cyclic or linear [8,9,10,11].

The structures that are produced may have two polarization states. The resonance stage is decentralized alternating between the two sulfurs in the SCS extremity. This decentralization provokes an approximation between both sulfurs with carbon reducing their facility to bind to another molecule due to system present a biggest stability. The other is the balanced stage, in which the polarization of electron occurs in one of the sulfurs SCS extremity, forming a double bond between C=S in another extremity. Because of this, the C-S extremity will have a negative charge and a bigger distance from the carbon, facilitating the capacity of interaction with other structures, including metals [9,10].

The type of chemical bond that formed among SCS extremity and others molecules may vary, according to the structural characteristics of the compounds, which may be of the monodentate type (a bond between the structure and the grouping), of the bidentate type (two symmetrical linkages between the structure and the grouping) or of the anisobidentate type (two asymmetrical distances). The SCS interaction shows an increase in bond strength, depending on the size of the metallic centre/chemical group and the alignment [10].

The ability of these structures to interact with metals gives an important biological value because a lot of proteins, molecules, and enzymes, which are present in their metallic compositions centres, are essential for these structures. Dithiocarbamates can be acting like chelators of metals, destabilizing the structure of these molecules and inhibiting their action [11]. Furthermore, as revised by Bond and Martin, the interaction with some metals is related to oxidative processes that are important mechanisms in the elimination of microorganisms [12].

Not only does the SCS extremity have a biological function, but also the amine present in this structure can interact with different substituents modulating responses and direct for specific targets. Furthermore, it acts producing reactive oxygen species (ROS), conferring an important biological activity for the dithiocarbamates [13,14].

Even if the general characteristics of the main chain are similar, each chemical group will have its own particular structure. Thus, small changes in structure, such as spatial changes of a certain segment, can generate changes in the function and behaviour of the formed derivatives, allowing for greater versatility for the biological application of new synthesized compounds [15].

The evaluation of compounds based on dithiocarbamates as potential new antimicrobials has been developed since the last century. In this scenario, several new molecules have been tested based on the principle of the basic structure and the derivation of these compounds, in order to show the different dithiocarbamate derivatives as antiprotozoal, anthelmintic, and antiviral agents. Therefore, compounds that are based on dithiocarbamates have been evaluated in different in vivo and in vitro models as potential new antimicrobials. Thus, the purpose of this review is to present the main uses of dithiocarbamate compounds as potential antitrypanosomatids-drugs, mainly for the therapeutic control of protozoa that are responsible for important human infectious diseases, such as Chagas disease, leishmaniasis, and African trypanosomiasis.

## 2. Main Chemical Groups Used in the Production of Dithiocarbamate Derivatives

Various synthetic compounds that are based on dithiocarbamates are produced because of their ability to form complexes with metals, as well as the ability to add different substituents to the final molecule structure. This attribute confers the classification of the dithiocarbamates based on the different chemical substituents attached to the molecule [15]. In this context, the main groups of chemical complexes based on dithiocarbamates are: (i) group 13 (III-A), which is generally formed by the use of halides with a parent of group 1 (I-A) or ammonium dithiocarbamate salt; (ii) group 14 (IV-A), which is the group of carbon compounds, esters of dithiocarbamic acid, which are also classified as organic compounds, which present applications that are directed towards the pharmaceutical field, agrochemicals, intermediates of organic synthesis, for drug and pro-drug formation, among others; (iii) group 15 (5-A), which is a well-studied group and demonstrates a wide variety of structures that can be employed in pharmacology, these compounds tend to act as an asymmetric chelator, in which a short binding between the metal and the sulfur atom; and, (iv) group 16 (VI-A) are complexes that interact with sulphates, resulting in organic compounds, such as thiurams, which can act as accelerators in vulcanization processes in the manufacture of compounds [16]. However, the main characteristic of group 16 is the change in the coordinates of metallic atoms. The interaction between metal and dithiocarbamate structure alters its spatial geometry and plane coordination relative to other molecular interaction models [17,18].

Several chemical compounds that belong to each of the chemical groups mentioned above might vary according to different characteristics, which may vary among chemical substituent in size, chain type (organic or inorganic), spatial geometry, types of binders, and functions, molecular targets. These compounds may act directly or indirectly on a variety of protozoa due to this chemical diversity [18,19] and are, therefore, evaluated as potential antiparasitic, as discussed below.

## 3. Pharmacological Evaluation of Dithiocarbamate as Potential Antitrypanosomatids-Drugs

### 3.1. Evaluation of Dithiocarbamate as Potential Anti-Trypanosoma Cruzi Drugs

*Trypanosoma cruzi* is a flagellate protozoan of the *Trypanosomatidae* family, which are responsible for Chagas disease, a neglected tropical disease that affects eight million people worldwide and causes more than 10,000 deaths annually. It is an endemic disease in several Latin American countries and is currently dispersed throughout the world due to migratory flows of populations [20,21].

The natural mechanism of transmission of Chagas disease occurs through vector transmission during blood repayment of previously infected triatomine insects. Other forms of transmission occur during blood transfusion, vertical transmission, organ transplants, and infected blood contacts [22,23]. Currently, the pharmacological treatment of Chagas disease consists of two main antiparasitic drugs, benznidazole and nifurtimox, drugs that are recognized by the World Health Organization for the treatment of infected individuals. These drugs have limitations on their use, mainly due to their high toxicity, and low or no pharmacological efficacy during the chronic treatment of Chagas disease [24].

**Figure 2:**
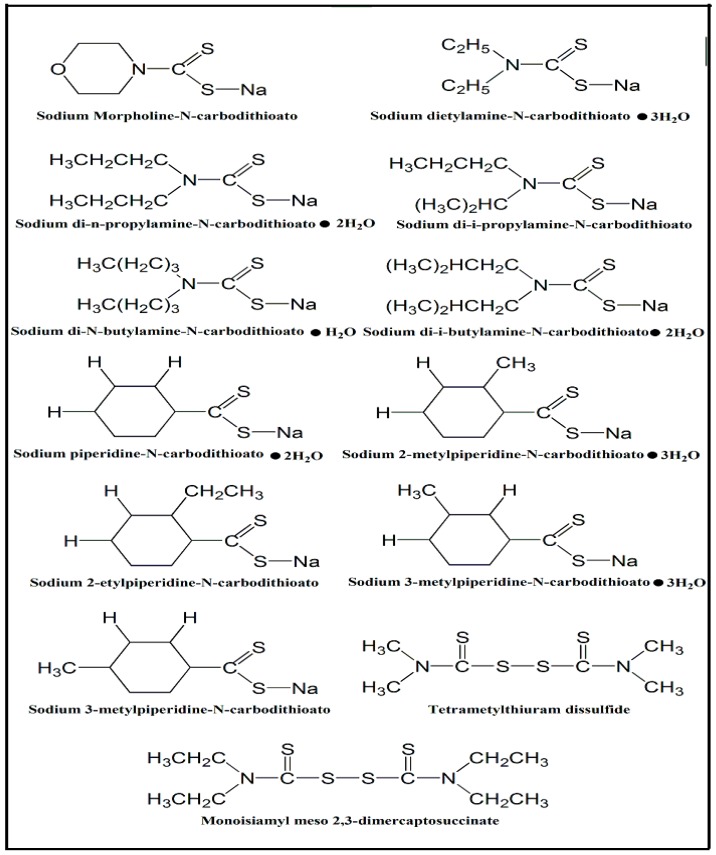
Compounds based on dithiocarbamate used as potential anti-*Trypanosoma cruzi* drugs. The figure represents the different structures of dithiocarbamates that showed antiparasitic activity. These compounds showed antiparasitic activity at a concentration of 5 µM during 72 h of treatment against the *T. cruzi* parasite. Increased production of reactive oxygen species seems to be the probable mechanism of action for these compounds to exhibit antiparasitic activity [26].

Several studies have demonstrated the ability of these compounds to inhibit the enzyme superoxide dismutase, an important enzyme in the oxidative system of many protozoa, due to the ability of dithiocarbamates to access intracellular compartments [25]. In this context, several compounds that are based on dithiocarbamates were synthesized (Figure 2). These compounds showed antiparasitic activity against *T. cruzi* when compared to the traditional benznidazole drug. The antiparasitic activity of dithiocarbamates is probably due to their ability to chelate metals, such as zinc, iron, and copper [26]. This biological activity in chelating metals allows for acting in other enzymes of the oxidation metabolism of protozoa, in addition to the enzyme superoxide dismutase, such as the carbonic anhydrase enzyme, which corresponds to another group of metalloproteinases with important function in parasite biology. These enzymes mainly participate in the redox metabolism of the parasite. Dithiocarbamate compounds may act on the metal centre of these enzymes by a chelation mechanism. Consequently, the parasite loses the ability to repair oxidative damage during parasitism in the host [27,28].

**Figure 3:**
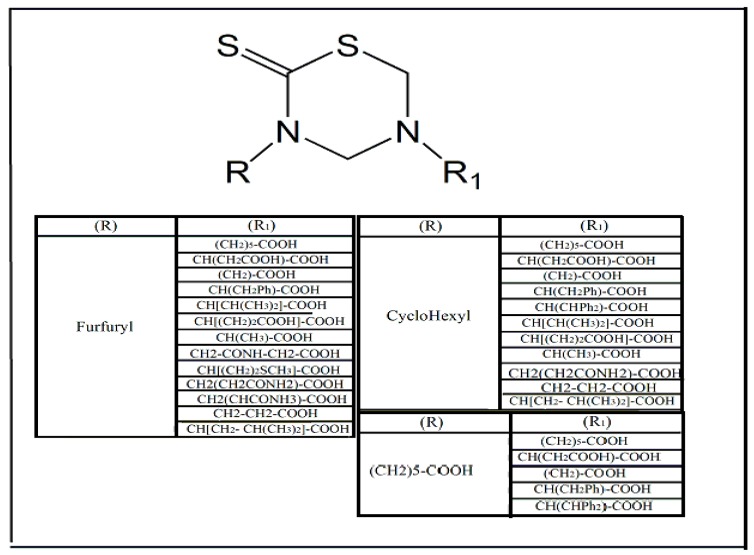
Thiadiazine-based synthetic compounds bound to dithiocarbamates with activity against *Trypanosoma cruzi*. The different substituents, represented by R1 and R2, may be attached to circular structures formed by thiadiazine and dithiocarbamate. The biological activity of these molecules appears to be related to their ability to inhibit the cysteine protease enzyme and increase oxidative damage in *T. cruzi* [31].

The exploration of new pharmacological targets for the development of new antiparasitic drugs is indispensable for the rational design of more specificity and low toxicity drugs [29]. Cysteine protease enzymes constitute a potential pharmacological target of protozoa that are responsible human infectious diseases. This class of enzymes chemically acts on thiol-imidazole conversion, which is an important process for parasite cell survival [30]. Compounds that are derived from thiadiazine have been shown to be enzymatic cysteine protease inhibitors of *Trichomonas vaginalis* and amastigote *T. cruzi* [30,31]. In this context, compounds that are derived from thiadiazine linked to dithiocarbamates were synthesized, resulting in the synthesis and characterization of 29 compounds with antiparasitic activity against *T. cruzi* (Figure 3). In addition, some of these compounds showed superior antiparasitic activity than nifurtimox, one of the therapeutic options during the pharmacological treatment of Chagas disease. The pharmacological activity that is attributed to these compounds is due to their ability to increase the production of reactive oxygen species, causing oxidative damage in the parasite and, consequently the loss of its viability [32].

### 3.2. Evaluation of Dithiocarbamate as Potential Anti-Leishmania Drugs

*Leishmania* sp. is a genus of flagellate protozoa that belongs to the family *Trypanosomatids*, which are etiological agents of zoonosis known as leishmaniasis, a neglected tropical disease that mainly affects populations of social and economic vulnerability. Leishmaniasis is a vector disease that can be transmitted by more than 20 species of *Leishmania* sp. Leishmaniasis is classified into two main clinical forms, visceral leishmaniasis and cutaneous leishmaniasis [33,34,35]. These are protozoa that present a complex life cycle, consisting of a phase in the invertebrate host of the genus *Phlebotomus* and another stage in the mammalian hosts (man, dog, wild animals, etc.) [36,37,38].

Pentavalent antimonials and amphotericin B are the major classes of compounds that are used in the pharmacological treatment of leishmaniasis. However, these drugs present high toxicity, which can cause hepatic, pancreatic, and renal impairment, consequently compromising patients’ quality of life during treatment [39,40]. Additionally, these drugs are responsible for causing oxidative damage in cells of humans due to their pharmacological action [41,42,43].

Previous studies with the chemical element osmium (III) have shown an expressive biological activity inhibiting various synthesis processes, such as DNA, RNA, and proteins, which are important for cell survival [44,45]. Due to this property, the metal ion osmium III was used by binding to dinitroimidazole and nitroimidazole, which were complexed to dithiocarbamate chains to increase antiparasitic activity against the protozoan *Leishmania donovani* (an etiological agent of leishmaniasis) and *T. cruzi*. In addition, this system presented a moderate cellular toxicity, as well as inhibited the action of several oxidative and energetic metabolism enzymes, such as succinate dehydrogenase, malate dehydrogenase, and pyruvate kinase of promastigotes *L. donovani* and epimastigote *T. cruzi* [46].

**Figure 4:**
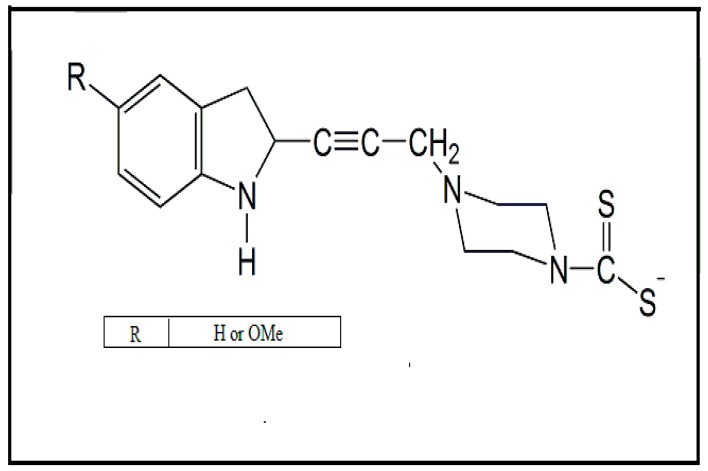
Chemical compounds synthesized from tryptophan with dithiocarbamates used as antiparasitic drugs against *Leishmania donovani* and *Trypanosoma cruzi*. In this figure is possible that different substituents (R: H or OMe) interact with the structure formed for tryptophan-dithiocarbamate. These structures are more efficient in inducing mitochondrial damage and consequently parasite death [47].

In other scenarios, analogous compounds of tryptophan have the ability to inhibit piroredoxin and the mitochondrial membrane complex II, or mitochondrial membrane complex FAD (Flavin and Adenine Dinucleotide), thereby triggering mitochondrial damage in *T. brucei* [47]. A series of dithiocarbamate-linked analogs produced compounds that showed antiparasitic efficacy at 30 μM against *L. donovani* and *T. cruzi* parasites. Moreover, these compounds, chemical structures, as represented in Figure 4, did not present cellular cytotoxicity in in vitro tests [48].

**Figure 5:**
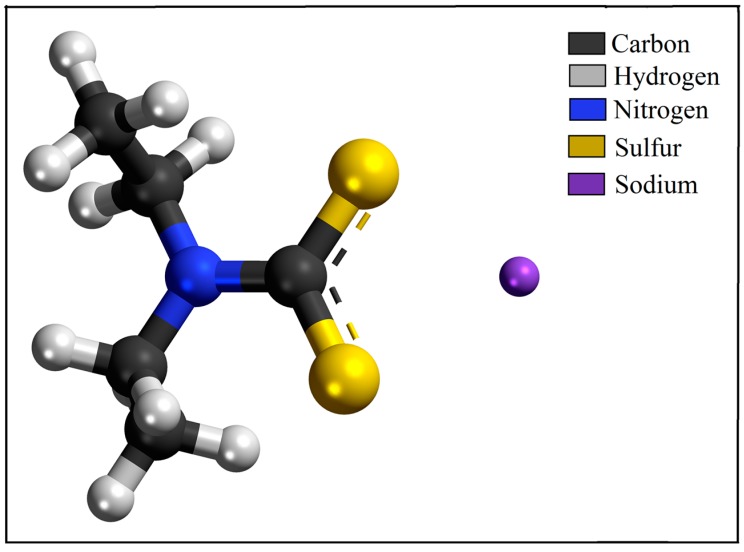
Sodium diethyldithiocarbamate (DETC) three-dimensional (3-D) chemical structure used as a potential anti-*Leishmania* sp. drug. DETC acts as a metal chelator and therefore inactivates enzymes essential for parasite survival [49,50].

Another approach to the antiparasitic activity of the compounds that are based on diethyldithiocarbamate was demonstrated using the sodium diethyldithiocarbamate compound (DETC—Figure 5) against the different forms of *L. amazonensis* and *L. braziliensis*, responsible for visceral leishmaniasis and cutaneous leishmaniasis, respectively.

Using several in vitro and in vivo models, the antiparasitic activity of DETC at low concentrations (1 mM and 2 mM) was demonstrated for the in vitro system in macrophages cells and for the in vivo system in murine models, respectively. DETC showed anti-*Leishmania* activity in both experimental models against the amastigote forms of *L. amazonensis* [49]. In this context, DETC was used to treat cutaneous lesion caused by *L. braziliensis*, where it was possible to observe a significant reduction in the overall parasitic load, as well as a decrease in the harmful cellular process that is triggered during infection [50]. In addition, DETC showed inhibitory activity for superoxide dismutase (SOD), which is an important enzyme that is involved in the redox metabolism of many protozoa of medical importance. This inhibitory activity is probably due to the interaction of the SCSNa end of the DETC molecule with the metal centre of the SOD enzyme, chelating the ferric centre, which is important for the catalytic activity [51].

**Figure 6:**
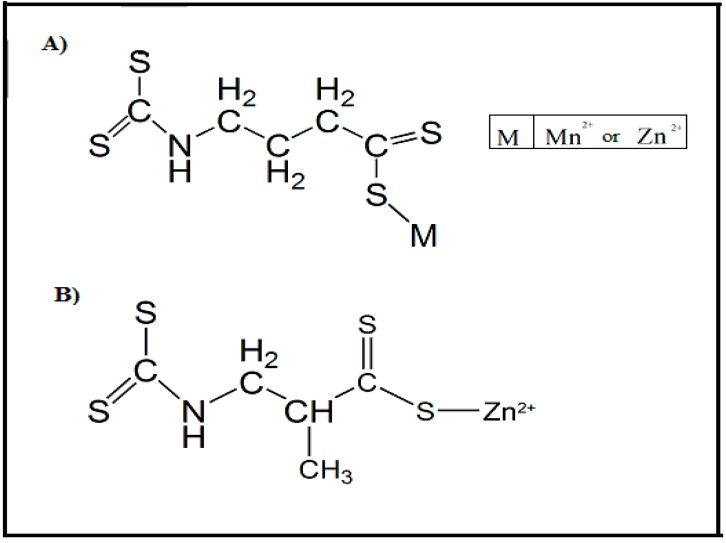
Synthetic dithiocarbamate derivatives bound to metal centres used as potential anti-*Leishmania* sp. drugs [52]. Two distinct chemical structures (A and B) that when interacting with metals (Mn^2+^ or Zn^2+^) exhibit enhanced antiparasitic activity against *L. donovani* parasites.

Various new compounds have been synthesized as potential anti-*Leishmania* drugs due to the versatility of the compounds based on dithiocarbamate. Compounds synthesized in metallic association with dithiocarbamates, named Maneb, Zineb and Propineb, were used against *L. donovani* [52]. The concentrations of 0.32 μM, 0.28 μM, and 0.14 μM were sufficient for eliminating 50% of the parasites. In addition, these compounds were found to have the ability to inhibit various isoforms of the carbonic anhydrase enzymes, an important component for parasite survival. Regarding the compounds shown in Figure 6, the bond between the dithiocarbamate chain and the metal is weak and easily dissociated. When this binding is broken, these free metals will interfere in oxidative processes and, therefore, stimulate the production of radical oxygen species (ROS) and intensify the response on parasite elimination [53,54,55].

### 3.3. Evaluation of Dithiocarbamate as Potential Anti-Trypanosoma Brucei Drugs

The protozoan *Trypanosoma brucei* is the etiological agent of African trypanosomiasis, also known as sleeping sickness. This protozoan is naturally transmitted through the bite of tsetse fly of the genus *Glossina* sp. Human African Trypanosomiasis (HAT) mainly affects 36 countries in sub-Saharan Africa, accounting for human infection with 100% mortality if not pharmacologically treated. The HAT is transmitted by two main subspecies of *T. brucei*: *T. b. gambiense* and *T. b. rhodesiense*, which are responsible for chronic and acute infections, respectively. Over 70 million people are in risk areas and situations in the context of HAT [56]. HAT therapy is extremely limited, because it has high toxicity and limited pharmacological efficacy. The main drugs that are used are melarsoprol, nifurtimox, eflornithine, and suramin [57].

**Figure 7:**
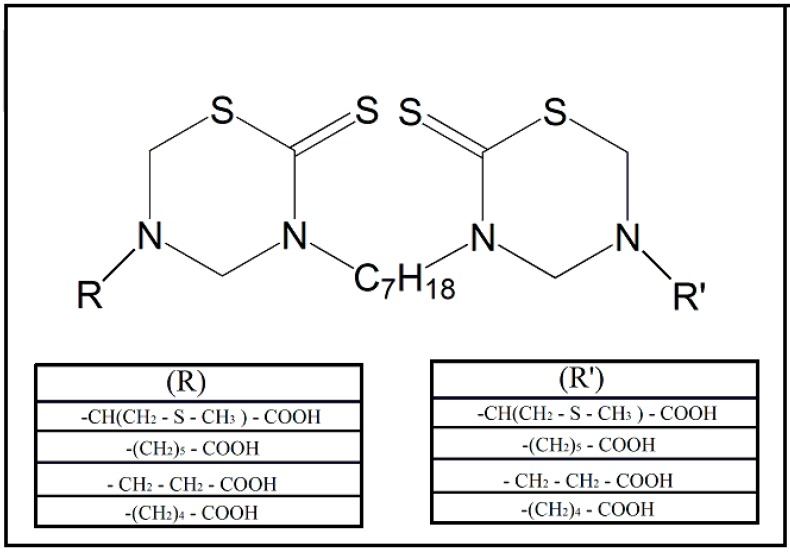
Synthetic dithiocarbamate derivatives bound to thiadiazone used as potential anti-*Trypanosoma brucei rhodesiense* drugs. The basic structures may receive different chemical substituents (R and R‘) that could influence the antiparasitic activity of the new synthesized compounds [62].

In the context of African trypanosomiasis, thiadiazine-based compounds, such as the tetrahydro-2H-1,3,5-thiadiazine-2-thione (THTT) derivatives, appear to be compounds with potential for use against *T. brucei*. This class of compounds stands out due to its inhibitory activity on the enzymes cysteine protease [58,59], along with the presence of the chemical group isothiocyanates [29]. This inhibitory activity occurs in a protic medium that is capable of cleaving the ring present in the THTT molecule [60]. On the other hand, due to the high solubility in lipophilic medium, its action in the enzymatic hydrolysis of several enzymes, present in some microorganisms, seems to be facilitated [61]. Various compounds that are based on BIS-THTT were synthesized due to these characteristics [62]. These compounds, as represented in the Figure 7, showed antiparasitic activity against *T. b. rhodesiense*, and a satisfactory selectivity index.

**Figure 8:**
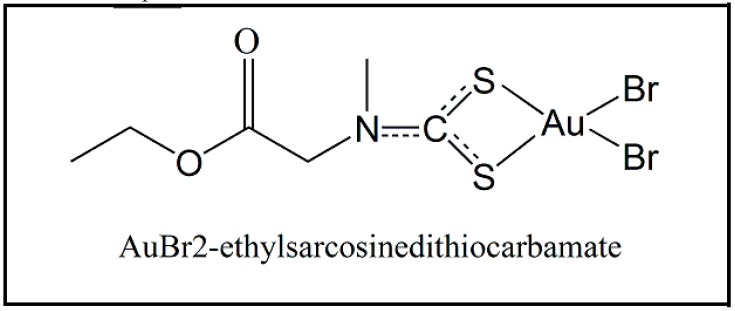
Synthetic dithiocarbamate derivatives bound to gold used as potential antiparasitic drugs against *Trypanosoma brucei*, *Leishmania infantum*, *Trypanosoma cruzi* and *Plasmodium falciparum*. The main biological activity attributed to this structure is related to its ability to induce oxidative stress and consequently parasite death [65].

Since 2010, a new class of gold-bound compounds has been shown to have different applications as antiparasitic drugs. These compounds appear to have an inhibitory action against thioredoxin reductase and thioredoxin-glutathione reductase enzymes, transcription factors, and some proteases that are present in various parasites [63,64]. In this context, gold-bound dithiocarbamate compounds, for example AuL12 (AuBr2-ethylsarcosinedithiocarbamate (EDTS)), as represented in the Figure 8, showed antiparasitic activity against *T. b. brucei* and had an IC50 of 2.06 μM [65]. In addition, this compound was also evaluated against different protozoan species, such as *T. cruzi, L. infantum, T. b. rhodesiense,* and *Plasmodium falciparum* (the etiological agent of malaria), and this compound showed a good parasite elimination performance in all cases, presented IC50 1.69, 2.83, 0.51, and 1.97, respectively. Therefore, since these enzymes are part of the antioxidative mechanism of the parasites, their inhibition process, by the action of gold metal, makes the parasites more susceptible to oxidative damages. However, the amine end of the dithiocarbamates is reactive and it stimulates the production of reactive oxygen species, thereby inducing the death of the parasites [65].

## 4. Final Considerations and Expectations

Several pharmacological applications of dithiocarbamate compounds have been popularized scientifically in recent decades. Due to the chemical and structural flexibility of these compounds, their use has been widely investigated as anti-carcinogenic agents [66,67,68,69,70,71,72,73,74,75,76,77], antibacterial [78,79], antiviral [80,81], antifungal [82,83], among others. In this review, we have presented and discussed the different applications of these compounds as potential antiparasitic compounds, more specifically as antitrypanosomatids-drugs against Chagas disease, leishmaniasis, and African trypanosomiasis. It is also important to note that some of these compounds have already been validated for their specific pharmacological targets in in vitro models [84,85,86,87,88,89,90,91,92,93].

Finally, dithiocarbamates are an important chemical compound that are to be considered in the rational development of antiparasitic drugs, especially in the context of neglected tropical diseases, an urgent need public health in many developing countries. Other important issues need to be resolved for the use of dithiocarbamates as potential new drugs, especially toxicological studies, definition of pharmacological targets, the parameters of chemical structure and biological activity, among others.
